# Acute decompensated right heart failure potentially triggered by multiple factors including pulmonary vasodilator removal during plasma exchange: a case report

**DOI:** 10.1186/s40981-025-00765-0

**Published:** 2025-01-27

**Authors:** Takayuki Toki, Kazuyuki Mizunoya, Misa Itabashi, Naoki Nishikawa, Koji Hoshino, Hitoshi Saito, Yuji Morimoto

**Affiliations:** Department of Anesthesiology and Critical Care Medicine, Hokkaido University Hospital, N14W5, Kita-ku, Sapporo, 060-8648 Japan

**Keywords:** Plasma exchange, Complications, Pulmonary vascular resistance, Pulmonary hypertension, Sildenafil

## Abstract

**Background:**

Plasma exchange (PE) removes high-molecular-weight substances and is sometimes used for antineutrophil cytoplasmic antibody-associated vasculitis (AAV) with alveolar hemorrhage. Hypotension during PE is rare, except in allergic cases. We report a case of shock likely caused by increased pulmonary vascular resistance (PVR) during PE.

**Case presentation:**

A 66-year-old man with pulmonary hypertension (PH) and glomerulonephritis was admitted with dyspnea. He had discontinued sildenafil prior to admission. Alveolar hemorrhage associated with AAV was suspected, and PE was performed. Soon after, he developed circulatory failure and hyperlactatemia. Echocardiography revealed right ventricular dilation, suggesting increased PVR. Inhaled nitric oxide (iNO) was administered, rapidly improving hyperlactatemia and oxygenation. The shock observed during PE was attributed to multiple factors, including the potential removal of sildenafil, which may have led to an increase in PVR.

**Conclusions:**

The shock was attributable to acute right heart failure caused by an exacerbation of PH, possibly due to sildenafil removal via PE, although other contributing factors could not be excluded.

## Background

Plasma exchange (PE) is a blood purification technique that removes high-molecular-weight pathogenic substances from plasma [1, 2].

Reports on PE complications indicate an incidence of adverse reactions ranging from 8.5 to 42.5% when fresh-frozen plasma (FFP) is used as the replacement fluid. However, hypotension with systolic blood pressure (SBP) below 95 mmHg occurs in only about 2.3% of cases, and SBP below 85 mmHg is observed in only 0.1–0.6% of cases [1, 2]. Most cases involve hypotension of allergic nature, and there are few reports suggesting that drug removal is the cause. Here, we report a case of shock that may have been caused by increased pulmonary vascular resistance (PVR) during PE.

## Case presentation

**Fig. 1:**
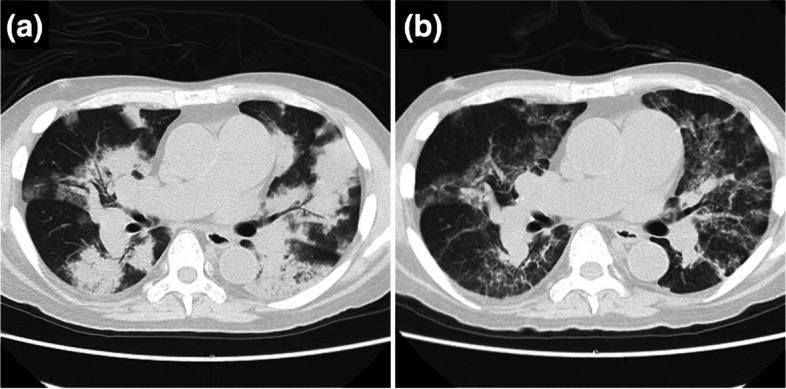
Chest computed tomography images. Computed tomography images showed bilateral diffuse ground-glass opacities and infiltrates upon admission (**a**). After 15 days, the ground-glass opacities and infiltrates showed improvement (**b**)

A 66-year-old Asian male traveler with pulmonary hypertension (PH), glomerulonephritis, and myelofibrosis was transported to our intensive care unit (ICU) due to sudden-onset dyspnea. Laboratory data indicated anemia (hemoglobin 6.8 g/dL), low platelet count (60,000/µL), kidney dysfunction (creatinine 1.82 mg/dL, blood urea nitrogen 58 mg/dL, and estimated glomerular filtration rate 30.4 mL/min), and a high inflammatory response (C-reactive protein 3.85 mg/dL). We contacted his family doctor in his home country but were unable to obtain detailed information about his PH history. Although his PH had been managed with sildenafil (20 mg, twice daily), he had been unable to take it since the night before admission due to severe dyspnea, and 18 h had passed since his last dose. On admission, his percutaneous oxygen saturation was 91% with 15 L/min of oxygen via face mask, temperature was 36.3 °C, noninvasive blood pressure was 196/90 mmHg, heart rate was 93 beats/min, and respiratory rate was 30 breaths/min. After tracheal intubation, the ratio of partial pressure of arterial oxygen to the fraction of inspired oxygen was 69.4. Computed tomography revealed bilateral diffuse ground-glass opacities and infiltrations (Fig. 1). Given his anemia and kidney dysfunction, alveolar hemorrhage associated with autoimmune diseases, such as systemic vasculitis, was considered. Alveolar hemorrhage and atelectasis were considered causes of hypoxemia. Airway pressure release ventilation (APRV) was initiated with the following settings: FIO_2_ 1.0, high pressure 25–30 cmH_2_O, high-pressure time 5.5 s, low pressure 0 cmH_2_O, and low-pressure time 0.5 s. However, there was no improvement in oxygenation. Routine use of PE for antineutrophil cytoplasmic antibody-associated vasculitis (AAV) is not generally recommended [3, 4]. However, some reports have demonstrated the beneficial effects of PE for AAV complicated by alveolar hemorrhage [5]. This case presented with rapidly progressive respiratory failure, which could have been fatal. CT findings and renal failure, suggestive of alveolar hemorrhage, prompted the semi-urgent decision to perform PE for suspected alveolar hemorrhage associated with AAV. We performed PE using 4800 mL of fresh-frozen plasma and started steroid pulse therapy with methylprednisolone at 1 g/day. Additionally, meropenem was administered as bacterial pneumonia could not be ruled out.

All oral medications were discontinued due to hemodynamic instability. As oxygenation did not improve with mechanical ventilation, bronchoscopy and bronchoalveolar lavage to confirm alveolar hemorrhage were not performed. Extracorporeal membrane oxygenation (ECMO) was avoided to prevent potential exacerbation of alveolar hemorrhage.

**Fig. 2:**
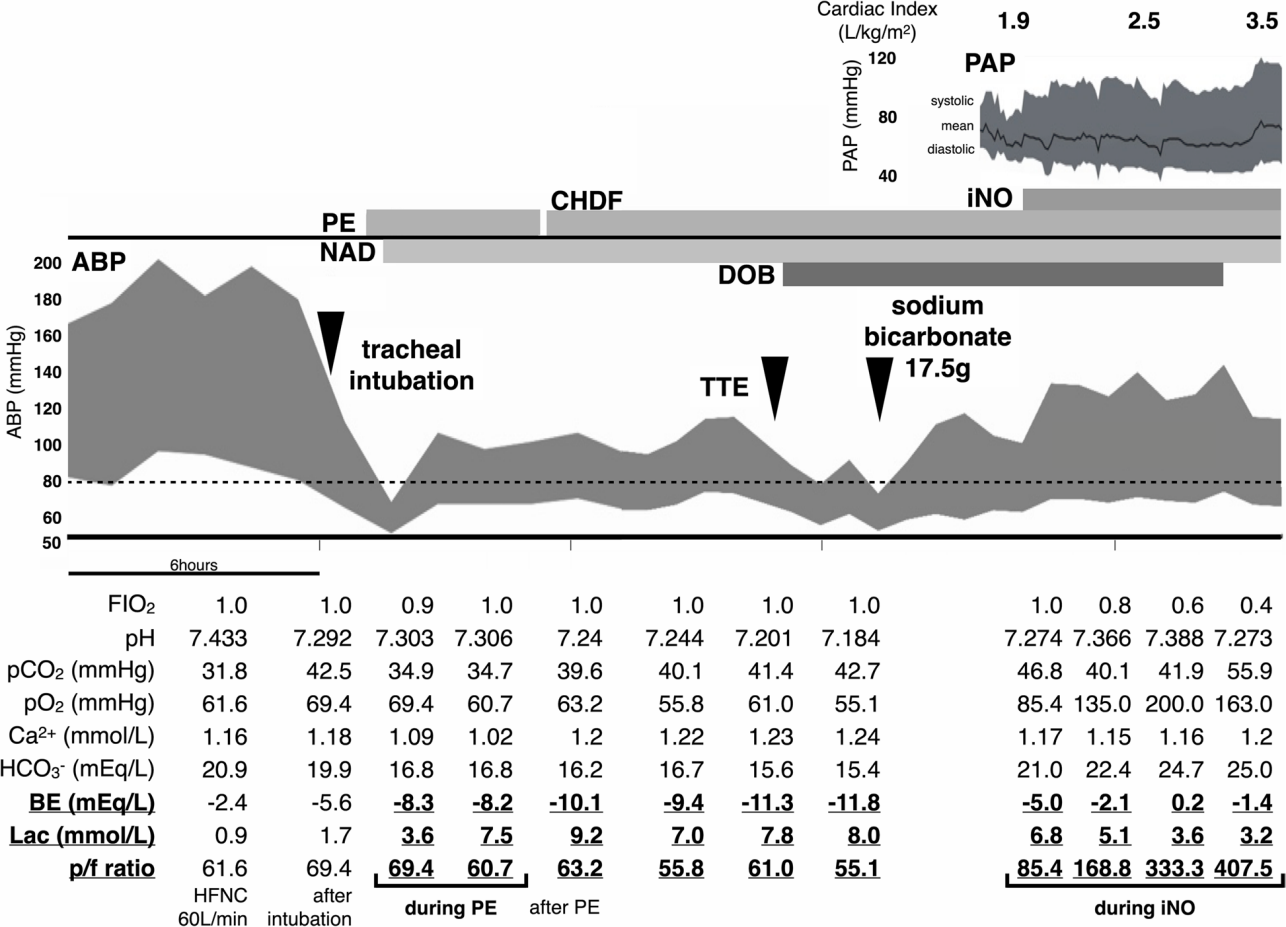
Clinical course and arterial blood gas analysis on day 1 and day 2. After begging plasma exchange, lactic acidosis worsened. In addition, oxygenation was inadequate. After beginning iNO, lactic acidosis and oxygenation improved, and arterial blood pressure increased. ABP, arterial blood pressure; PAP, pulmonary artery pressure; PE, plasma exchange; iNO, inhaled nitric oxide; NAD, noradrenaline; DOB, dobutamine; FIO_2_, inspired oxygen fraction; pCO_2_, partial pressure of arterial carbon dioxide; pO_2_, partial pressure of arterial oxygen; HCO_3_^−^, bicarbonate ion; Lac, lactate; p/f, partial pressure of arterial oxygen/inspired oxygen fraction; HFNC, high-flow nasal cannula

Shortly after starting PE, the patient developed hypotension with systemic blood pressure (SBP) dropping below 80 mmHg, accompanied by worsening lactic acidosis (Fig. 2). No skin symptoms or other allergic reactions were observed. Continuous noradrenaline infusion was required to maintain blood pressure. Although we suspected an overdose of sedatives and reduced the propofol dose, noradrenaline infusion had to be continued. After completing PE, severe lactic acidosis persisted. Continuous renal replacement therapy was started to manage the acidosis; however, no improvement was observed, indicating that circulatory failure played a significant role. Transthoracic echocardiography showed a dilated right ventricle and a collapsed left ventricle, suggesting right ventricular failure. Continuous dobutamine infusion was started, and the mean pulmonary arterial pressure (PAP) was 54 mmHg measured by a pulmonary artery catheter placement. We diagnosed the patient with acute right heart failure due to severe PH, and inhaled nitric oxide (iNO) was administered to decrease PVR. After iNO administration, both circulatory insufficiency and hypoxemia showed significant improvement. Additionally, the cardiac index measured using HemoSphere® (Edwards Lifesciences, Tokyo) and systemic blood pressure increased, enabling the tapering of noradrenaline and dobutamine. Central venous pressure decreased rapidly from 15 to 12 mmHg after starting iNO, though PAP remained elevated initially (Fig. 2). Blood test results obtained upon ICU admission, available after performing PE, revealed that all autoantibodies indicative of AAV were negative. Consequently, alveolar hemorrhage due to AAV was deemed unlikely. By day 14, the mean PAP gradually decreased to 42 mmHg as respiratory function improved, fluid was removed, and macitentan was administered. Initially, macitentan was chosen instead of restarting sildenafil due to concerns that sildenafil’s platelet aggregation inhibition might worsen alveolar hemorrhage. Procalcitonin on day 7 was 13.20 ng/mL, so meropenem was continued, although no pathogens were identified in the sputum.

The patient was successfully weaned off the ventilator and iNO by day 9 and transferred out of the ICU on day 14. On day 15, information from the previous medical provider revealed a treatment history for pulmonary artery hypertension, with a recent mean PAP of 49 mmHg. As the PAP had returned to a similar level and there was no evidence of alveolar hemorrhage, sildenafil was resumed, while macitentan was gradually tapered and discontinued. The patient was discharged on day 33 with supplemental oxygen via nasal cannula.

## Discussion

In this case, prolonged circulatory insufficiency and hypoxemia occurred during PE and continued afterward. Initially, the cause of the shock was unclear. However, transthoracic echocardiography and right heart catheterization suggested acute right heart failure due to increased PVR. Immediately after initiation of iNO, a potent pulmonary vasodilator, circulatory failure, and hypoxemia rapidly improved.

The exacerbating factors of PH in this case include pulmonary infection, hypoxemia, and positive pressure ventilation. The patient’s hemodynamic condition markedly worsened after the initiation of tracheal intubation and PE. Positive pressure ventilation can increase intrathoracic pressure and PVR, potentially leading to right heart failure [6]. In this case, since hypotension was observed immediately after tracheal intubation and starting mechanical ventilation with APRV mode, we cannot exclude the effect of positive airway pressure on right heart failure. However, the deterioration in lactate acidosis and oxygenation immediately after PE suggested that the PE had a significant impact. Additionally, hemodynamic improvement was observed immediately after initiating iNO while continuing APRV under the same conditions, which suggests that factors other than positive pressure ventilation had a major effect.

Although no direct evidence links PE to the worsening of PH, we finally hypothesize that the withdrawal of pulmonary vasodilators, specifically sildenafil, during PE may have contributed to the acute right heart failure observed in this case. However, other factors likely played significant roles. The physiological effects of tracheal intubation and positive pressure ventilation, both of which are known to increase PAP and exacerbate right ventricular dysfunction in patients with preexisting PH, warrant consideration. Furthermore, imaging revealed extensive pulmonary infiltration, suggesting significant alveolar strain and an associated rise in PVR. In this case, the last dose of sildenafil was taken approximately 18 h before admission, and based on the half-life of sildenafil which is 3.26 to 4.52 h [7, 8], it is likely that the plasma concentration had dropped significantly by the time PE was initiated. However, in elderly patients with renal impairment, such as this case, the pharmacokinetics of sildenafil may differ, potentially leading to prolonged effective plasma concentrations [7, 8]. This raises the possibility that sildenafil levels were further reduced by PE, which removes high-molecular-weight substances bound to plasma proteins. While this hypothesis aligns with the patient’s clinical deterioration during PE, the lack of direct evidence for sildenafil removal or its concentration changes limits the certainty of this conclusion.

In general, molecular weight, protein-binding ratio (PBR), and volume of distribution (Vd) are important factors for drug removal during hemodialysis [9]. Drugs with a high PBR and Vd are generally not easily removed by hemodialysis or hemofiltration. However, PE removes plasma from the blood and replaces it with FFP or albumin solution [10]. Thus, drugs with a high PBR, though not a high Vd, may be removed by PE. Sildenafil, a selective phosphodiesterase type 5 inhibitor, has a PBR of 96–97% [11] and a Vd of approximately 105 L [12]. Sildenafil has been reported to be minimally removed by hemodialysis [13]; however, no studies have examined its pharmacokinetics during PE. A review of PE’s impact on drug pharmacokinetics indicates moderate removal of cardiovascular drugs with high PBR and Vd, such as verapamil, propranolol, and amlodipine [14]. Therefore, sildenafil may also be partially removed by PE. We proposed that the increase in PVR observed in this case may have been multifactorial, with contributions from poor lung condition due to infection, pulmonary vascular strain induced by positive pressure ventilation, and potentially reduced sildenafil levels due to its removal by PE. Severe hypoxia and acidemia likely compounded these effects. While sildenafil removal by PE is a plausible contributing factor, further research, including pharmacokinetic studies during PE, is necessary to substantiate this hypothesis.

Inhaled nitric oxide (iNO) selectively reduces PVR in ventilated lung areas without causing systemic vasodilation [15]. Although several recent reports describe successful treatment of adult PH cases with iNO [16, 17], other studies indicate that iNO may be ineffective or even harmful in adults with PH [18, 19]. Currently, limited evidence exists regarding the effectiveness of iNO for adult PH. In our case, however, iNO led to rapid improvements in lactic acidosis and hypoxemia, indirectly supporting the hypothesis that increased PVR caused prolonged lactic acidosis and respiratory failure during PE.

## Conclusions

This case highlights a rare instance of prolonged shock during and after PE, which responded to iNO. We concluded that shock was attributable to acute right heart failure caused by PH exacerbation, possibly due to the removal of sildenafil by PE. However, other contributing factors could not be excluded.

## Data Availability

Not applicable.

## Abbreviations

PE: Plasma exchange
SBP: Systolic blood pressure
PH: Pulmonary hypertension
PAP: Pulmonary artery pressure
PVR: Pulmonary vascular resistance
ICU: Intensive care unit
FFP: Fresh-frozen plasma
iNO: Inhaled nitric oxide
PBR: Protein-binding ratio
Vd: Volume distribution
